# Whole Exome Sequencing Identifies a Novel Pathogenic RET Variant in Hirschsprung Disease

**DOI:** 10.3389/fgene.2018.00752

**Published:** 2019-01-14

**Authors:** Wei Wu, Li Lu, Weijue Xu, Jiangbin Liu, Jun Sun, Lulu Zheng, Qingfeng Sheng, Zhibao Lv

**Affiliations:** Department of General Surgery, Shanghai Children’s Hospital, Shanghai Jiao Tong University, Shanghai, China

**Keywords:** whole exome sequencing, Hirschsprung disease, RET variant, minor allele frequencies, bioinformatics

## Abstract

Hirschsprung disease is a birth defect characterized by complete absence of neuronal ganglion cells from a portion of the intestinal tract. To uncover genetic variants contributing to HSCR, we performed whole exome sequencing on seven members of an HSCR family. With the minor allele frequency (MAF) calculated by gnomAD, we finally filtered a total of 1,059 rare variants in this family (MAF < 0.1%). With the mode of inheritance and pathogenicity scores by bioinformatics tools, we identified an in-frameshift variant p.Phe147del in *RET* as the disease-causing variant. Further analysis revealed that the in-frameshift variant may function by disrupting the glycosylation of RET protein. To our knowledge, this is the first study to report the in-frameshift variant p.Phe147del in RET responsible for heritable HSCR.

## Introduction

Hirschsprung disease (HSCR) is a congenitally genetic disorder of the enteric nervous system (ENS) characterized by complete absence of neuronal ganglion cells from a portion of the intestinal tract. The incidence of HSCR is approximately 1 in 5,000 live births, which varies among different ethnic groups (Parisi and Kapur, 2000). HSCR can be classified into short-segment HSCR (S-HSCR), long-segment HSCR (L-HSCR), and total colonic aganglionosis (TCA) based on the length of the aganglionic segment (Lantieri et al., 2006). Treatment options for HSCR include surgical treatment with resection of the aganglionic segment and reconstitution of the intestinal passage after the first year of life, following bridging therapy with colostomy (Bachmann et al., 2015).

The mode of inheritance of HSCR vary from dominant with reduced penetrance or recessive in familial cases to a more complex, non-Mendelian mode of inheritance in the sporadic cases (Amiel et al., 2008). So far, several genes have been found to contribute to HSCR, such as *RET* (Tomuschat and Puri, 2015), *ECE1* (Vohra et al., 2007), *EDN3* (Sanchez-Mejias et al., 2010), *EDNRB* (Sanchez-Mejias et al., 2010), *GDNF* (Eketjall and Ibanez, 2002), *NRTN* (Doray et al., 1998), *SOX10* (Lecerf et al., 2014), *PHOX2B* (Fernandez et al., 2013), and *KIAA1279* (Amiel et al., 2008). With the exception of the *RET* proto-oncogene that is responsible for approximately 50% of familial and up to 15% of sporadic cases, other HSCR genes only account for a small proportion of the cases (Amiel et al., 2008). *RET* encodes a transmembrane tyrosine kinase receptor that, during development of specific neuronal cell lineages, transduces extracellular signals for cell growth and differentiation. The mechanism of HSCR caused by loss-of-function mutations in RET is highly dependent on their location in the protein (So et al., 2011). For example, mutations affecting the intra-cytoplasmatic domain could impair the kinase activity required for proper signal transduction, altering either the catalytic function, the stability of the enzyme structure or the binding of transduction effectors (Hyndman et al., 2013). In contrast, mutations of the extracellular domain (ECD) can affect RET function through a number of different mechanisms, such as lack of ligand binding, and impairment of protein folding (Kjaer and Ibanez, 2003). However, these mechanisms are mostly revealed for missense, nonsense, frameshift, and splicing mutations, and the pathogenicity of in-frameshift variants is underestimated by previous studies.

In the present study, we collected an HSCR family with four affected and three unaffected members. To uncover the novel pathogenic genes or variants, we performed whole exome sequencing on seven family members. With the filtering steps by minor allele frequency (MAF), the mode of inheritance, co-segregation, and pathogenicity scores by the bioinformatics tools, we identified a novel in-frameshift variant p.Phe147del in RET as the disease-causing variant, which may function by disrupting RET N-glycosylation. To our knowledge, this is the first study to report the p.Phe147del in RET as a disease-causing variant for heritable HSCR.

## Results

### Clinical Features of the HSCR Cases

**FIGURE 1:**
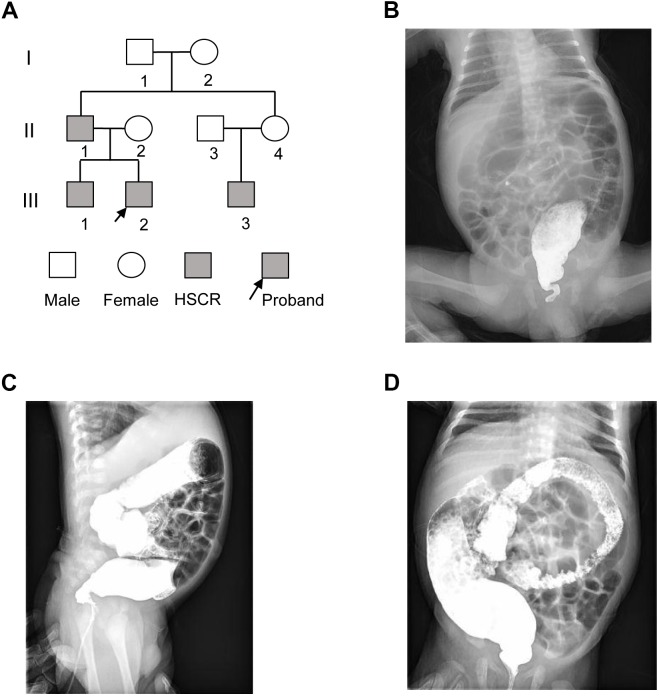
The pedigree of three generations of the HSCR family. **(A)** Pedigree of the three-generation, Chinese family with four affected individuals. Squares indicate males, and circles represent females. Black and white symbols represent affected and unaffected individuals, respectively. The proband is indicated by an arrow. **(B–D)** The colon X-ray images of the proband at three different views.

The proband (III-2) was a 2-month-old Chinese boy who had the symptoms of abdominal distension and vomiting after birth (Figure 1A). The proband was diagnosed as Hirschsprung disease by barium enema examination (Figures 1B–D), which showed typical symptoms of congenital megacolon. In detail, we observed that the ganglion cells were present in dilated segment, but not detected in narrow segment of mucosa of intestinal wall and myenteric nerve plexus by microscopic image-based histologic examination. Unmyelinated nerve fiber and Schwann cells were increased in the narrow segment. Further family history survey revealed that the proband’s father (II-1), older brother (III-1), and cousin (III-3) had the similar symptoms (Figure 1A). The proband as well as his older brother and cousin was diagnosed as short-form and long-form aganglionosis based on the length of aganglionosis, respectively. Unfortunately, the length of aganglionosis was unclear due to loss of medical record. All the patients were cured by radical operation.

### Identification of Rare Variants in Coding or Splicing Regions

**FIGURE 2:**
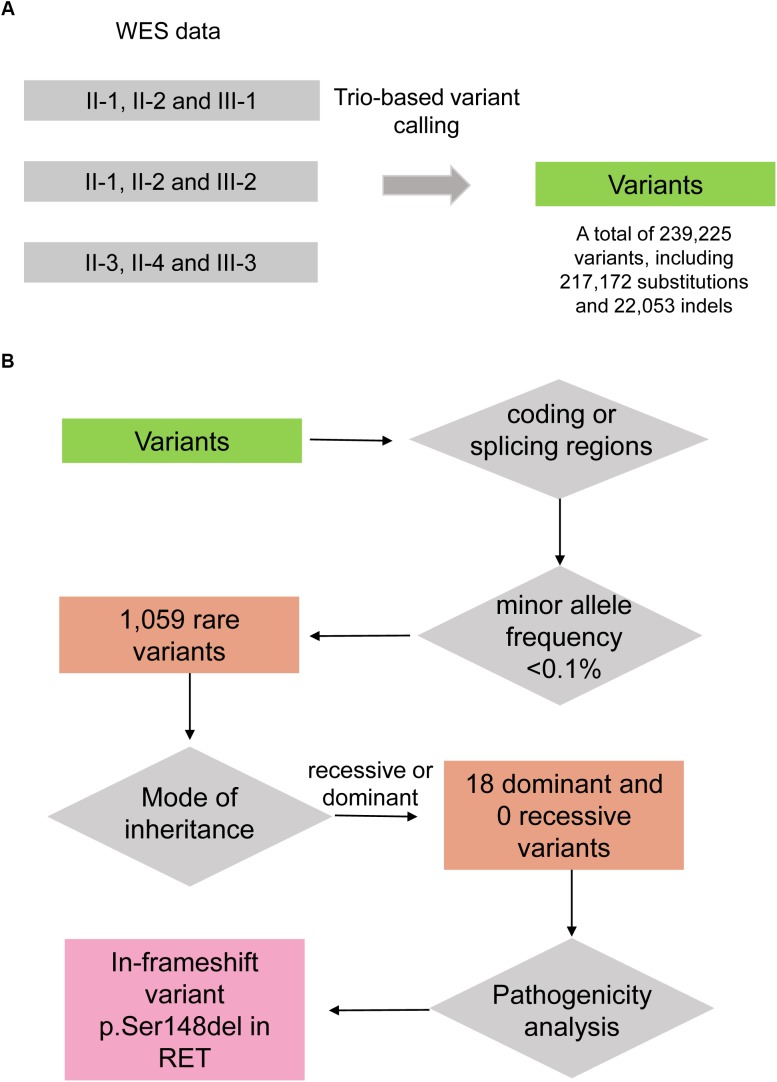
The workflow of discovering the disease-causing variant for the HSCR family. **(A)** Variants are called by VarScan with the trio-based calling mode. **(B)** The disease-causing variant is identified by filtering steps including falling within coding/splicing regions, the minor allele frequency (MAF) in healthy population, mode of inheritance, and pathogenicity analysis.

To uncover the genetic variants contributing to HSCR, we performed whole exome sequencing on the seven family members, including II-1, II-2, II-3, II-4, III-1, III-2, and III-3. Variants were called by VarScan (Koboldt et al., 2012) with the trio-based mode. The steps of genetic variant analysis were illustrated in Figure 2. In total, we detected 239,225 variants at which at least one family member had an allele that varied from the reference genome, including 217,172 substitutions and 22,053 indels (insertion and deletion) (Figure 2A). For the three affected boys (III-1, III-2, and III-3), the Mendelian errors were estimated about 1.10, 1.43, and 1.33%, suggesting that the variants were high reliable by the trio-based variant calling strategy.

As the heritable Hirschsprung disease was rare in the population, the pathogenic variants were more likely to be rare in healthy population. To identify the rare variants, we firstly obtained their MAFs from gnomAD database (Lek et al., 2016). By excluding the non-coding and synonymous variants, we finally filtered a total of 1,059 rare variants in this family (MAF < 0.1%). As the Hirschsprung disease could be inherited by autosomal dominant and recessive patterns (Amiel et al., 2008), autosomal dominant and recessive variants were considered. Among the homozygous variants and biallelic variants, no recessive variants were shared by the four patients. Notably, under the hypothesis of autosomal dominant inheritance, the unaffected female, II-4, may be a carrier of the pathogenic variant due to incomplete penetrance. Finally, we identified 18 dominant variants (Figure 2B), including 14 missenses, 1 nonsense, 2 frameshifts, and 1 in-frameshift variants, co-segregated in the three boys.

### Identification of Pathogenic Candidate for HSCR

To identify pathogenic variants, we evaluated the pathogenicity of the 18 dominant variants using bioinformatics tools, such as SIFT (Ng and Henikoff, 2003), PolyPhen (Adzhubei et al., 2013), MutationTaster (Schwarz et al., 2010), M-CAP (Jagadeesh et al., 2016), DDIG-in (Zhao et al., 2013), and SIFT-indel (Hu and Ng, 2013) (Supplementary Table S1). The 14 missense variants were filtered using the pathogenic scores in SIFT (≤0.05), PolyPhen (≥0.957), MutationTaster (‘disease causing’), and M-CAP (>0.025), and *CAPN9*, *GLYCTK*, and *DRD5* were recognized. Moreover, *RET*, *FANCI*, and *CALN1* were identified by the two pathogenicity prediction algorithms for indels, DDIG-in and SIFT-indel. The nonsense variant in *NPHP3* was recognized as potentially pathogenic by MutationTaster and DDIG-in. In summary, seven genes, including *CAPN9*, *GLYCTK*, *DRD5*, *NPHP3*, *FANCI*, *CALN1*, and *RET*, were pathogenic candidates by the bioinformatics tools.

**FIGURE 3:**
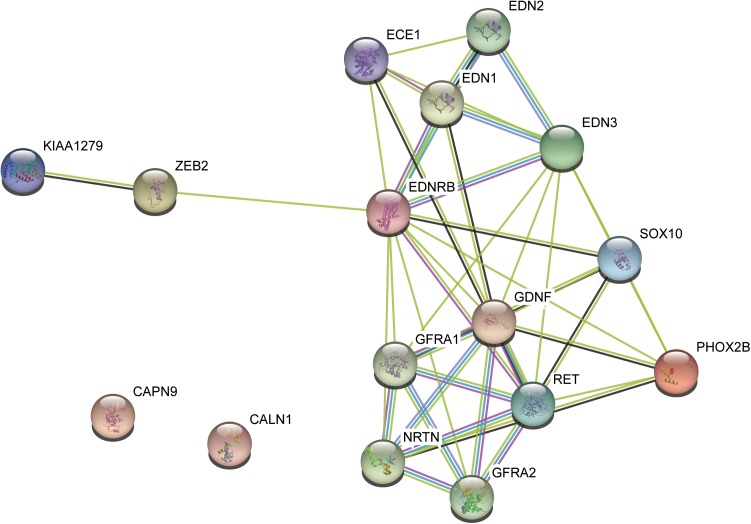
The protein–protein interaction subnetwork of two pathogenic candidates and nine known pathogenic genes.

To further evaluate the relationship between the variants and HSCR, we performed literature review about the seven pathogenic candidates. We found one in-frameshift variant p.Phe147del in RET, the most commonly observed pathogenic gene for HSCR. The remaining genes, such as *GLYCTK* (Sass et al., 2010)*, DRD5* (Daly et al., 1999)*, NPHP3* (Bergmann et al., 2008), and *FANCI* (Mehta and Tolar, 1993), were well-characterized pathogenic genes for some other rare diseases with recessive mode of inheritance or multi-gene diseases, such as D-glyceric aciduria, attention deficit-hyperactivity disorder, Meckel syndrome, and Fanconi Anemia. However, these genes were excluded due to recessive inheritance of their associated diseases. To further clarify the implications of *CALN1* and *CAPN9* in HSCR, we mapped the *RET*, *CALN1* and *CAPN9*, combined with some known HSCR pathogenic genes, including, *ECE1*, *EDN3*, *EDNRB*, *GDNF*, *NRTN*, *SOX10*, *PHOX2B*, and *KIAA1279*, to protein–protein interaction (PPI) network curated in STRING database (Szklarczyk et al., 2015). *CALN1* and *CAPN9* were observed to connect with none of these known genes directly or indirectly within five nodes, suggesting that the two genes may not be pathogenic for HSCR (Figure 3). Only RET connected with the known pathogenic genes in the PPI network, in particular, which was also a known HSCR gene. The result indicated that the in-frameshift variant p.Phe147del in RET was pathogenic for the HSCR family.

### Potential Impact of the In-Frameshift Variant on RET Protein Function

**FIGURE 4:**
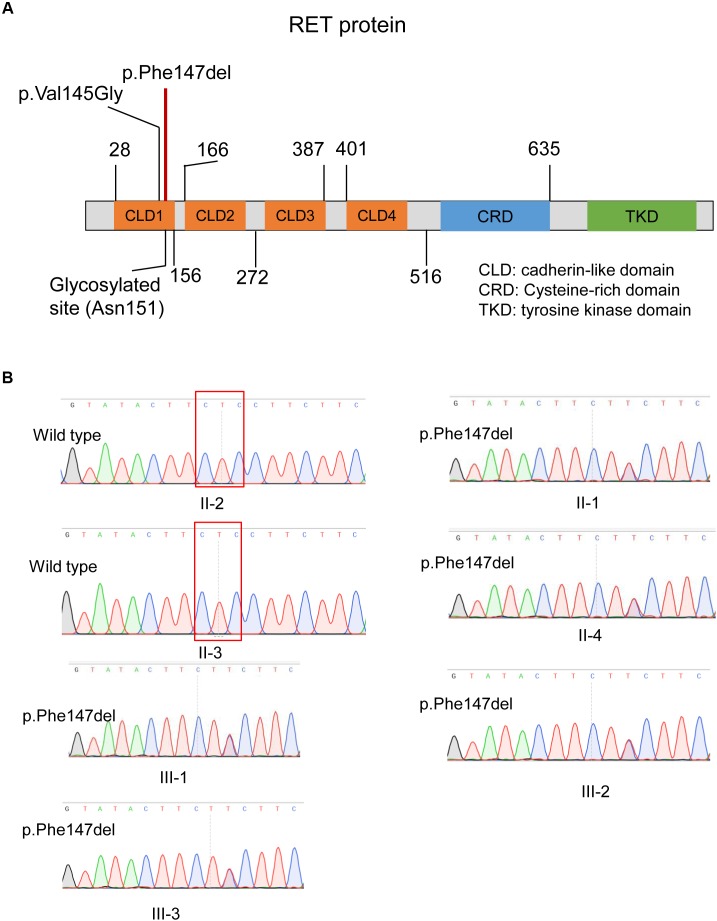
The in-frameshift disease-causing variant in the RET protein structure and validation of this variant by Sanger sequencing. **(A)** The in-frameshift disease-causing variant p.Phe147del in RET is located within the CLD1 domain. **(B)** The in-frameshift variant in RET (p.Phe147del) in four patients and one unaffected carrier, and two unaffected members with wild genotype are validated by Sanger sequencing. The deleted three bases were enclosed by the red box.

As illustrated in Figure 4A, the ECD of RET was composed of four cadherin-like domains (CLD1-4), and cysteine-rich domain (CRD). We found the in-frameshift variant p.Phe147del was located within CLD1. Previous study (Leon et al., 2012) reported that a disease-causing mutation at pVal145Gly in *RET*, which was close to the in-frameshift variant p.Phe147del, could disrupt RET N-glycosylation, giving us a hint that the in-frameshift variant may also function by disrupting RET N-glycosylation. To determine the consequence of the in-frameshift variant p.Phe147del on RET glycosylation, we predicted the N-glycosites of wild-type and p.Phe147del RET proteins using GlycoEP (Chauhan et al., 2013), a webserver for predicting potential N- and O-glycosites in protein sequence. Moreover, the N-glycosites of p.Val145Gly was also predicted as a positive control. Finally, the sites of Asn151, Asn834 and Asn1084 were predicted to be glycosylated in wild-type RET protein (GlycoEP score > 0.85). However, Asn151, which is closest glycosylated site to the two mutants, p.Phe147del and p.Val145Gly, was predicted to be not glycosylated in the two mutant RET proteins (Table 1). The result indicated that the in-frameshift variant p.Phe147del could function by disrupting RET N-glycosylation.

**Table 1:** The glycosylated sites predicted by GlycoEP for RET proteins with wild-type, and p.Phe147del and p.Val145Gly mutants.

RET protein	Asn151	Asn834	Asn1084
Wild-type	+	+	+
p.Phe147del	-	+	+
p.Val145Gly	-	+	+


*+, glycosylated site (GlycoEP score > 0.85)*.

*-, non-glycosylated site (GlycoEP score < 0.85)*.

### Validation of Pathogenic Variant by Sanger Sequencing

We validated the two variants by Sanger sequencing (Figure 4B). Our results demonstrated that the four patients carried the in-frameshift variant p.Phe147del in RET. Moreover, we also confirmed our hypothesis that the pathogenic variant carrier II-4 was unaffected due to incomplete inheritance. In accordance with the genotyping by whole exome sequencing, the other family members, II-2 and II-3, were wild genotypes. The result indicated that whole exome sequencing was efficient to identify pathogenic variants for monogenic inherited diseases.

## Materials and Methods

### Ethics Statement

The present study was approved by the Ethics Committee of the Children’s Hospital of Shanghai Jiao Tong University, Shanghai, China, and was conducted according to the principles expressed in the Declaration of Helsinki. Participants and/or their legal guardians involved in this study gave a written informed consent prior to inclusion in the study.

### Sample Collection

The present study included DNA samples from four patients (II-1, III-1, III-2, and III-3) and three unaffected family members (II-2, II-3, and II-4) as shown in Figure 1A. Genomic DNA samples were obtained with written informed consent. TIAN amp Blood DNA Kit (Tiangen Biotech, Co., Ltd., Beijing) was used for extracting genomic DNA from blood samples.

### Reads Mapping and Variants Calling

Paired-end reads of 300 bp (150 bp at each end) from whole exome sequencing were mapped to UCSC human reference genome (GRCh37/hg19 assembly) using BWA (Li and Durbin, 2010) version 0.7.7-r441 ‘mem’ mode with default options followed by removal of PCR duplicates and low-quality reads (BaseQ < 20). The bam files were then sorted and indexed by samtools (Li et al., 2009), and were converted as three-sample mpileup format for each parents-offspring trio. Variant calling was performed by VarScan (Koboldt et al., 2012) software^1^ using version 2.3.7 of Trios mode.

### Variant Annotation and Prioritization

We used the ANNOVAR (Wang et al., 2010) software to annotate the MAF in gnomAD (Lek et al., 2016) database, variant pathogenicity scores by SIFT (Ng and Henikoff, 2003), PolyPhen (Adzhubei et al., 2013), MutationTaster (Schwarz et al., 2010), M-CAP (Jagadeesh et al., 2016), RefSeq gene and the consequences on protein, such as missense, frameshift, in-frameshift, stop-gain, and splicing. For the indels, we used DDIG-in (Zhao et al., 2013) and SIFT-indel (Hu and Ng, 2013) to evaluate their pathogenicity. Rare variants (MAF < 0.1% in Asian cohort) were filtered based on the gnomAD (Lek et al., 2016) database.

### Prediction of Glycosylated Sites

The glycosylated sites for RET proteins with wild-type, and p.Phe147del and p.Val145Gly mutants were predicted by GlycoEP (Chauhan et al., 2013)^2^ with standard predictor, a webserver for predicting potential N- and O-glycosites in protein sequence. The prediction was performed based on Average Surface Accessibility (ASA+BPP) with threshold score 0.85.

## Discussion

Hirschsprung disease is a birth defect characterized by complete absence of neuronal ganglion cells from a portion of the intestinal tract. In the present study, we performed whole exome sequencing on seven members of the HCSR family to identify the disease-causing gene. Microscopic image-based histologic examination of the proband’s diseased tissue also observed the absence of neuronal ganglion cells in narrow segment of mucosa of intestinal wall and myenteric nerve plexus.

To uncover genetic variants contributing to HSCR, we identified 100s of 1000s variants in seven members of the HSCR family using whole exome sequencing data. The lower Mendelian error demonstrated that trio-based variant calling was an effective strategy for family-based sequencing. As the heritable Hirschsprung disease was rare in the population, the disease-causing variants were more likely to be rare in healthy population. With the MAF calculated by gnomAD, we finally filtered a total of 1,059 rare variants in this family (MAF < 0.1%). In addition to MAF in healthy population, the dominant and recessive inheritance modes of HSCR were also considered in this family. In general, the autosomal recessive genes may be altered by two compound heterozygous variants, or one homozygous variant. However, we did not detect any recessive variants based on this assumption. For the assumption of autosomal dominant inheritance, the unaffected female, II-4, must be a carrier of the pathogenic variant due to incomplete penetrance, in accordance with the previous studies (Parisi and Kapur, 2000; Belknap, 2002). Moreover, the pathogenic variant must be co-segregated in the four patients and the carrier. The filtering steps by MAF, mode of inheritance and co-segregation could greatly exclude non-pathogenic variants.

To accurately identify the pathogenic variants, we further performed pathogenicity analysis on the 18 dominant variants. Generally, the algorithms evaluating the pathogenicity of single nucleotide substitutions and indels were different. Specifically, among the single nucleotide substitutions, the missense variants were mostly evaluated by SIFT, PolyPhen, MutationTaster and M-CAP, while the nonsense variants were evaluated by MutationTaster and DDIG-in. On the other side, we evaluated the pathogenicity of indels, frameshift and in-frameshift variants, using DDIG-in (Zhao et al., 2013) and SIFT-indel (Hu and Ng, 2013). Among the 18 dominant variants, seven genes or variants, including *CAPN9*, *GLYCTK*, *DRD5*, *NPHP3*, *FANCI*, *CALN1*, and *RET*, were predicted as pathogenic candidates by these algorithms. To our knowledge, GLYCTK (Sass et al., 2010), DRD5 (Daly et al., 1999), NPHP3 (Bergmann et al., 2008), and FANCI (Mehta and Tolar, 1993), were well-characterized pathogenic genes for some other rare diseases with recessive mode of inheritance or multi-gene diseases, such as D-glyceric aciduria, attention deficit-hyperactivity disorder, Meckel syndrome, and Fanconi Anemia. However, these genes were excluded due to recessive inheritance of their associated diseases. In addition, CAPN9 and CALN1 are thought to be associated with gastric cancer (Yoshikawa et al., 2000) and schizophrenia (Schizophrenia Psychiatric Genome-Wide Association Study (GWAS) Consortium, 2011), respectively. To further narrow down the gene list that may contribute to HSCR, we performed literature review and mapped the pathogenic candidates to protein–protein interaction network. Apart from *RET*, the literature review and PPI network analysis successfully excluded the other six genes. Notably, p.Phe147del in *RET* was a novel pathogenic variant in HSCR based on the curation by ClinVar database (Landrum et al., 2018). Finally, the in-frameshift variant p.Phe147del in RET, the most commonly observed pathogenic gene for HSCR, was identified as the pathogenic variant.

To further examine the functional impact of the in-frameshift variant p.Phe147del in *RET* on the occurrence of the disease, we mapped the variant to RET protein structure. It is well-recognized that variants locating within specific functional domains or protein translation modification sites could alter the protein conformation, protein–ligand binding, or protein–protein interaction. In this study, the in-frameshift variant p.Phe147del in RET was located within the CLD1 domain. We accessed the Uniprot database, and found that the five amino acids adjacent to the in-frameshift variant were only characterized to be glycosylated, not be phosphorylated or methylated. Further literature investigation also accorded with the annotations by Uniprot database. *RET* encodes a transmembrane receptor, which is composed of ECD, transmembrane domain, and intracellular tyrosine kinase domain. Particularly, the CLD1 domain belongs to the ECD. Further analysis of the consequence of p.Phe147del variant on the RET protein revealed that this in-frameshift variant may disrupt glycosylation of RET protein, which may be the cause of HSCR in this family.

Compared with previous studies, our study focused on the cases from familial HSCR. The pathogenic genes for familial HSCR including RET, EDNRB and EDN3, exhibited high penetrance. However, for the sporadic cases with Hirschsprung disease, like the report by Tang et al. (2018), some novel pathogenic or susceptibility genes, such as PLD1, had reduced penetrance, indicating that the penetrance of pathogenic genes was higher in familial HSCR than the sporadic Hirschsprung disease. The sporadic Hirschspring disease may be caused by additive effect of the susceptibility genes and environmental factors.

In reality, the lack of experimental validation is a major concern about this research. However, we conducted systematic bioinformatics analysis to demonstrate the pathogenicity and functionality of the variant in this family. To our knowledge, this is the first study to report the in-frameshift variant p.Phe147del in RET responsible for heritable HSCR. In conclusion, the systematic analysis of this study not only improved understanding of the causes of this disease, but also was useful for clinical and prenatal diagnosis.

## Author Contributions

ZL and WW collaborated in the conception and design of the present study. LZ and LL collected and assembled the data. WW and QS were involved in data analysis and interpretation. All authors contributed to writing the manuscript and approved the final version.

## Conflict of Interest Statement

The authors declare that the research was conducted in the absence of any commercial or financial relationships that could be construed as a potential conflict of interest.

## Footnotes

1 http://varscan.sourceforge.net/

2 http://crdd.osdd.net/raghava/glycoep/

## Supplementary Material

TABLE S1 The evaluation of the pathogenicity of the 18 dominant variants using bioinformatics tools.

## References

[B1] Adzhubei I. Jordan D. M. Sunyaev S. R. (2013). Predicting functional effect of human missense mutations using PolyPhen-2. *Curr. Protoc. Hum. Genet.* Chapter 7:Unit7.20. 10.1002/0471142905.hg0720s76 23315928 PMC4480630

[B2] Amiel J. Sproat-Emison E. Garcia-Barcelo M. Lantieri F. Burzynski G. Borrego S. (2008). Hirschsprung disease, associated syndromes and genetics: a review. *J. Med. Genet.* 45 1–14. 10.1136/jmg.2007.053959 17965226

[B3] Bachmann L. Besendorfer M. Carbon R. Lux P. Agaimy A. Hartmann A. (2015). Immunohistochemical panel for the diagnosis of Hirschsprung’s disease using antibodies to MAP2, calretinin. GLUT1 and S100. *Histopathology* 66 824–835. 10.1111/his.12527 25123159

[B4] Belknap W. M. (2002). The pathogenesis of Hirschsprung disease. *Curr. Opin. Gastroenterol.* 18 74–81. 10.1097/00001574-200201000-00013 17031234

[B5] Bergmann C. Fliegauf M. Bruchle N. O. Frank V. Olbrich H. Kirschner J. (2008). Loss of nephrocystin-3 function can cause embryonic lethality, Meckel-Gruber-like syndrome, situs inversus, and renal-hepatic-pancreatic dysplasia. *Am. J. Hum. Genet.* 82 959–970. 10.1016/j.ajhg.2008.02.017 18371931 PMC2427297

[B6] Chauhan J. S. Rao A. Raghava G. P. (2013). In silico platform for prediction of N-, O- and C-glycosites in eukaryotic protein sequences. *PLoS One* 8:e67008. 10.1371/journal.pone.0067008 23840574 PMC3695939

[B7] Daly G. Hawi Z. Fitzgerald M. Gill M. (1999). Mapping susceptibility loci in attention deficit hyperactivity disorder: preferential transmission of parental alleles at DAT1. DBH and DRD5 to affected children. *Mol. Psychiatry* 4 192–196. 10.1038/sj.mp.4000510 10208453

[B8] Doray B. Salomon R. Amiel J. Pelet A. Touraine R. Billaud M. (1998). Mutation of the RET ligand, neurturin, supports multigenic inheritance in Hirschsprung disease. *Hum. Mol. Genet.* 7 1449–1452. 10.1093/hmg/7.9.1449 9700200

[B9] Eketjall S. Ibanez C. F. (2002). Functional characterization of mutations in the GDNF gene of patients with Hirschsprung disease. *Hum. Mol. Genet.* 11 325–329. 10.1093/hmg/11.3.325 11823451

[B10] Fernandez R. M. Mathieu Y. Luzon-Toro B. Núñez-Torres R. González-Meneses A. Antiñolo G. (2013). Contributions of PHOX2B in the pathogenesis of Hirschsprung disease. *PLoS One* 8:e54043. 10.1371/journal.pone.0054043 23342068 PMC3544660

[B11] Hu J. Ng P. C. (2013). SIFT Indel: predictions for the functional effects of amino acid insertions/deletions in proteins. *PLoS One* 8:e77940. 10.1371/journal.pone.0077940 24194902 PMC3806772

[B12] Hyndman B. D. Gujral T. S. Krieger J. R. Cockburn J. G. Mulligan L. M. (2013). Multiple functional effects of RET kinase domain sequence variants in Hirschsprung disease. *Hum. Mutat.* 34 132–142. 10.1002/humu.22170 22837065

[B13] Jagadeesh K. A. Wenger A. M. Berger M. J. Guturu H. Stenson P. D. Cooper D. N. (2016). M-CAP eliminates a majority of variants of uncertain significance in clinical exomes at high sensitivity. *Nat. Genet.* 48 1581–1586. 10.1038/ng.3703 27776117

[B14] Kjaer S. Ibanez C. F. (2003). Intrinsic susceptibility to misfolding of a hot-spot for Hirschsprung disease mutations in the ectodomain of RET. *Hum. Mol. Genet.* 12 2133–2144. 10.1093/hmg/ddg227 12915470

[B15] Koboldt D. C. Zhang Q. Larson D. E. Shen D. McLellan M. D. Lin L. (2012). VarScan 2: somatic mutation and copy number alteration discovery in cancer by exome sequencing. *Genome Res.* 22 568–576. 10.1101/gr.129684.111 22300766 PMC3290792

[B16] Landrum M. J. Lee J. M. Benson M. Brown G. R. Chao C. Chitipiralla S. (2018). ClinVar: improving access to variant interpretations and supporting evidence. *Nucleic Acids Res.* 46 D1062–D1067. 10.1093/nar/gkx1153 29165669 PMC5753237

[B17] Lantieri F. Griseri P. Ceccherini I. (2006). Molecular mechanisms of RET-induced Hirschsprung pathogenesis. *Ann. Med.* 38 11–19. 10.1080/07853890500442758 16448984

[B18] Lecerf L. Kavo A. Ruiz-Ferrer M. Baral V. Watanabe Y. Chaoui A. (2014). An impairment of long distance SOX10 regulatory elements underlies isolated Hirschsprung disease. *Hum. Mutat.* 35 303–307. 10.1002/humu.22499 24357527

[B19] Lek M. Karczewski K. J. Minikel E. V. Samocha K. E. Banks E. Fennell T. (2016). Analysis of protein-coding genetic variation in 60,706 humans. *Nature* 18 285–291. 10.1038/nature19057 27535533 PMC5018207

[B20] Leon T. Y. So M. T. Lui V. C. Hofstra R. M. Tam P. K. Ngan E. S. (2012). Functional analyses of RET mutations in Chinese Hirschsprung disease patients. *Birth Defects Res. A Clin. Mol. Teratol.* 94 47–51. 10.1002/bdra.22863 22131258

[B21] Li H. Durbin R. (2010). Fast and accurate long-read alignment with burrows-wheeler transform. *Bioinformatics* 26 589–595. 10.1093/bioinformatics/btp698 20080505 PMC2828108

[B22] Li H. Handsaker B. Wysoker A. Fennell T. Ruan J. Homer N. (2009). The sequence alignment/map format and SAMtools. *Bioinformatics* 15 2078–2079. 10.1093/bioinformatics/btp352 19505943 PMC2723002

[B23] Mehta P. A. Tolar J. (1993). *Fanconi Anemia* eds Adam M. P. Ardinger H. H. Pagon R. A. Seattle, WA: GeneReviews.

[B24] Ng P. C. Henikoff S. (2003). SIFT: predicting amino acid changes that affect protein function. *Nucleic Acids Res.* 31 3812–3814. 10.1093/nar/gkg509 12824425 PMC168916

[B25] Parisi M. A. Kapur R. P. (2000). Genetics of Hirschsprung disease. *Curr. Opin. Pediatr.* 12 610–617. 10.1097/00008480-200012000-00017 11106284

[B26] Sanchez-Mejias A. Fernandez R. M. Lopez-Alonso M. Antinolo G. Borrego S. (2010). New roles of EDNRB and EDN3 in the pathogenesis of Hirschsprung disease. *Genet. Med. Off. J. Am. Coll. Med. Genet.* 12 39–43. 10.1097/GIM.0b013e3181c371b0 20009762

[B27] Sass J. O. Fischer K. Wang R. Christensen E. Scholl-Bürgi S. Chang R. (2010). D-glyceric aciduria is caused by genetic deficiency of D-glycerate kinase (GLYCTK). *Hum. Mutat.* 31 1280–1285. 10.1002/humu.21375 20949620

[B28] Schizophrenia Psychiatric Genome-Wide Association Study (GWAS) Consortium (2011). Genome. (-)wide association study identifies five new schizophrenia loci. *Nat. Genet.* 43 969–976. 10.1038/ng.940 21926974 PMC3303194

[B29] Schwarz J. M. Rodelsperger C. Schuelke M. Seelow D. (2010). Mutation taster evaluates disease-causing potential of sequence alterations. *Nat. Methods* 7 575–576. 10.1038/nmeth0810-575 20676075

[B30] So M. T. Leon T. Y. Cheng G. Tang C. S. Miao X. P. Cornes B. K. (2011). RET mutational spectrum in Hirschsprung disease: evaluation of 601 Chinese patients. *PLoS One* 6:e28986. 10.1371/journal.pone.0028986 22174939 PMC3235168

[B31] Szklarczyk D. Franceschini A. Wyder S. Forslund K. Heller D. Huerta-Cepas J. (2015). STRING v10: protein-protein interaction networks, integrated over the tree of life. *Nucleic Acids Res.* 43 D447–D452. 10.1093/nar/gku1003 25352553 PMC4383874

[B32] Tang C. S. Li P. Lai F. P. Fu A. X. Lau S. T. So M. T. (2018). Identification of genes associated With Hirschsprung disease, based on whole-genome sequence analysis, and potential effects on enteric nervous system development. *Gastroenterology* 155 1908.e5–1922.e5. 10.1053/j.gastro.2018.09.012 30217742

[B33] Tomuschat C. Puri P. (2015). RET gene is a major risk factor for Hirschsprung’s disease: a meta-analysis. *Pediatr. Surg. Int.* 31 701–710. 10.1007/s00383-015-3731-y 26164711

[B34] Vohra B. P. Planer W. Armon J. Fu M. Jain S. Heuckeroth R. O. (2007). Reduced endothelin converting enzyme-1 and endothelin-3 mRNA in the developing bowel of male mice may increase expressivity and penetrance of Hirschsprung disease-like distal intestinal aganglionosis. *Dev. Dyn.* 236 106–117. 10.1002/dvdy.21028 17131407

[B35] Wang K. Li M. Hakonarson H. (2010). ANNOVAR: functional annotation of genetic variants from high-throughput sequencing data. *Nucleic Acids Res.* 38:e164. 10.1093/nar/gkq603 20601685 PMC2938201

[B36] Yoshikawa Y. Mukai H. Hino F. Asada K. Kato I. (2000). Isolation of two novel genes, down-regulated in gastric cancer. *Jpn J. Cancer Res. Gann.* 91 459–463. 10.1111/j.1349-7006.2000.tb00967.x 10835488 PMC5926377

[B37] Zhao H. Yang Y. Lin H. Zhang M. Mort D. N. Cooper Y. (2013). DDIG-in: discriminating between disease-associated and neutral non-frameshifting micro-indels. *Genome Biol.* 14:R23. 10.1186/gb-2013-14-3-r23 23497682 PMC4053752

